# Online Masquerade: Redesigning the Internet for Free Speech Through the Use of Pseudonyms

**DOI:** 10.1111/japp.12342

**Published:** 2018-09-12

**Authors:** Carissa Véliz

**Affiliations:** ^1^ Uehiro Centre for Practical Ethics, Wellcome Centre for Ethics and Humanities, Faculty of Philosophy Christ Church University of Oxford Oxford OX11PT

## Abstract

Anonymity promotes free speech by protecting the identity of people who might otherwise face negative consequences for expressing their ideas. Wrongdoers, however, often abuse this invisibility cloak. Defenders of anonymity online emphasise its value in advancing public debate and safeguarding political dissension. Critics emphasise the need for identifiability in order to achieve accountability for wrongdoers such as trolls. The problematic tension between anonymity and identifiability online lies in the desirability of having low costs (no repercussions) for desirable speech and high costs (appropriate repercussions) for undesirable speech. If we practice either full anonymity or identifiability, we end up having either low or high costs in all online contexts and for all kinds of speech. I argue that free speech is compatible with instituting costs in the form of repercussions and penalties for controversial and unacceptable speech. Costs can minimise the risks of anonymity by providing a reasonable degree of accountability. Pseudonymity is a tool that can help us regulate those costs while furthering free speech. This article argues that, in order to redesign the Internet to better serve free speech, we should shape much of it to resemble an online masquerade.

Posting online is dangerous. An online harasser murdered one of Japan's most prominent bloggers in June 2018.^1^ A few months before that, human rights activist Issa al‐Nukheifi was sentenced to 6 years in prison because of tweets that were critical of the Saudi government. Between 2010 and 2015, 2,500 Londoners were arrested over ‘offensive’ posts online.^2^ Countless people, often from vulnerable populations, have suffered online harassment after exposing their views online. If all netizens were anonymous, none of these speakers would have faced such serious repercussions for their posts.

Negative consequences of online postings can range from suffering others’ disdain, to being the target of bullying, losing one's job, liberty, or even one's life. Anonymity serves to advance certain goals in society, such as free speech, by protecting the identity of people who might otherwise be in danger. This invisibility cloak, however, can be abused. People can use the protection afforded by anonymity to attack others, or engage in other kinds of misbehaviour. Champions of anonymity emphasise its value in advancing public debate, protecting political dissension, and furthering due process. Critics emphasise the need for accountability and the interest of the public in knowing the identity of speakers.

At a first glance, the values on both sides of the debate seem equally weighty. Free and open debate, public dissension, and due process seem just as important as accountability and verifiability. However, when we look at the downsides of anonymity versus those of identifiability, I argue that, more often than not, it is easier to correct the issues that anonymity brings with it than it is to address the problems created by identifiability. I argue that free speech is compatible with instituting costs in the form of repercussions and penalties for improper speech. Costs can minimise the risks of anonymity, and pseudonymity can help us regulate those costs. This article argues that we should shape much of the Internet to resemble an online masquerade.

Section 1 reviews reasons for and against anonymity. In Section 2, I weigh two options against each other: practicing identifiability while trying to achieve the benefits of anonymity through means different from anonymity, and practicing anonymity while trying to obtain the benefits of identifiability through other means. The objective is to explore the ways of securing our most important aims through means other than anonymity and identifiability, so that we might profit from the benefits without the disadvantages that anonymity and identifiability bring. In Section 3, I defend the view that not all speech should be equally costly: some kinds of speech should be low‐cost, and others should be high‐cost. Section 4 proposes pseudonymity as a way to regulate costs in speech. Section 5 responds to the objection of deception. Section 6 deals with the objection of censorship. Section 7 emphasises the importance of contracts in protecting identity.

## 1 Anonymity: Reasons for and Against

Someone is anonymous when others have not identified her through traits that only attach to her. Each person's identity is constituted by interrelated traits – much like a network of dots. Some traits, such as name and address, are quite specific to each person. Other traits, such as gender, are not specific enough to identify someone as a unique person. Knowing enough non‐specific traits, however, can lead to knowledge of specific traits. Thus, William Thackeray, in one of his *Roundabout Papers*,* On Being Found Out*, tells the story of a priest who mentions at a dinner party that the first confession he received was from a murderer. Some minutes later, a marquis joins the group and greets the priest affectionately, introducing himself to the other guests as the priest's first penitent.^3^

As the story illustrates, an anonymous person loses her anonymity with regard to another when the observer ‘joins the dots’ through either knowledge of identifiable traits, such as name and address, or through knowledge of enough non‐identifiable traits that permit inferring her unique identity (and the sensitive information that typically attaches to identity).

Anonymity is sometimes involuntary, as when an ordinary person walks in the midst of strangers in a crowded city street without the intention of being anonymous. Often, anonymity is sought after and intentionally brought about to fulfil two general purposes: furthering some desirable action (e.g. the publication of an idea) and protecting agents (e.g. protecting authors from possible retaliation).^4^

Writing is the natural home of anonymity, as it was the process of writing that first facilitated it on a large scale. In prehistory, speech was intimately tied to the speaker's identity. With people living in small communities, the opportunities for anonymity that big cities have brought about were largely absent – only lone travellers could be strangers. With the advent of writing, however, the possibility of anonymity became widespread.

Speaking one's mind has always been a risky business. If others judge the message to be offensive or threatening to the status quo, they can respond defensively. Examples in history abound: from Socrates’ death sentence for impiety and corruption of the youth in 399bc, to Galileo's house arrest in the 17^th^ century for defending the view that the Earth revolves around the Sun, and Turkey's current persecution of academics in the wake of last year's military coup.^5^ It is therefore unsurprising that writers have often made use of anonymity to protect themselves. In fact, most writing before the Renaissance was anonymous.^6^ John Locke's *Two Treatises on Government*; Hamilton, Madison and Jay's *The Federalist Papers,* Marx and Engels’ *Communist Manifesto* and Kierkegaard's works are among the many masterpieces that have been published anonymously and that otherwise might have never seen the light of day.

### 1.1 Reasons for Anonymity

Protection from harm is the weightiest and most common reason to seek anonymity. But there are at least two other significant reasons in favour of anonymous writing. First, some writers such as Kierkegaard believe that the writing process itself is influenced by the thought of authorship and identifiability.^7^ According to this view, the writing will be better if the author is as little burdened as possible by his own identity and fears about his reputation.

Second, anonymity in writing allows for the work to be evaluated on its own merits, without interfering judgements about the identity of the author. Charlotte Brontë and George Eliot, among many other women, did not want their gender to be known in order to avoid prejudice.^8^ Virginia Woolf wrote: ‘I would venture to guess that Anon, who wrote so many poems without signing them, was often a woman’.^9^ Proponents of anonymity in authorship contend that both literary talent and arguments should be judged independently of the author's identity or reputation.

Academic double‐blind peer review constitutes a case in point. Manuscripts are not accepted or rejected on the basis of the author's name, but on the basis of the work's merit, thus contributing to fairness and the pursuit of rigour and truth. Double‐blind peer review is designed to protect both authors and reviewers from holding a grudge against the other, thereby allowing the freedom for authors to write possibly unpleasant or audacious views, and for reviewers to criticise articles and recommend rejection when appropriate.^10^ Anonymity protects the process of fair review by shielding authors from discrimination on the part of reviewers, and reviewers from possible reprisals or undue feelings of debt from authors.

In the context of the Internet, anonymity protects netizens from each other, from corporations, and from government surveillance. As the District Court Judge said in a leading US case on online regulation, the Internet ‘is the most participatory form of mass speech yet developed’.^11^ Anonymity encourages the expression of more views, particularly unorthodox views, by more people – particularly minorities.

This effect points to the wide interest we have in anonymity. It is not only speakers and authors who have a stake in free speech being encouraged, but society at large. If anonymity were not an option, citizens would miss out on views that would otherwise not be expressed. John Stuart Mill famously argued in *On Liberty* that people have an interest in having access to different opinions:

… the peculiar evil of silencing the expression of an opinion is, that it is robbing the human race; posterity as well as the existing generation […]. If the opinion is right, they are deprived of the opportunity of exchanging error for truth: if wrong, they lose, what is almost as great a benefit, the clearer perception and livelier impression of truth, produced by its collision with error.^12^

In a similar vein, Scanlon has argued that individuals have a right to consider all arguments available for possible courses of action.^13^

Anonymity protects the expression of valuable views in the form of political protest and whistleblowing, among other outlets. Whistle‐blowers help society by exposing relevant and suppressed information, typically about powerful people or institutions. They inform the general population about controversial and thitherto unknown practices. Because they are speaking out against power, more often than not, whistle‐blowers face retaliation if their identity is known.^14^ Similarly, political protest, among other functions, helps to alert governments and individuals about neglected areas of concern, including unfair or dangerous government policies. Protestors can be social innovators, changing public opinion and inspiring people to take action in bettering their society.^15^

### 1.2 Reasons Against Anonymity

Although anonymity fulfils an important role in promoting desirable speech and protecting agents from unfair and negative reactions from others, it can also facilitate undesirable speech and protect agents from deserved and fair reactions. The possible negative effects of anonymity were already apparent to Plato. In Book II of the *Republic*, Glaucon argues that, if people could have access to a magical artefact, the ring of Gyges, which could make them invisible to others, no one would be so virtuous as to resist the temptation of performing bad acts such as theft and murder.^16^ More than two thousand years later, the moral problem remains, only aggravated by technology. On the Internet, the ring of Gyges ceases to be a thought experiment. In the virtual world, anonymity can serve as an invisibility ring available to anyone. To the extent that more of our lives are spent online, anonymity is becoming more of a problem, and it lies behind some of the most alarming trends on the Internet.

Anonymity can encourage irresponsible writing – impulsive, offensive or even violent language, as well as inaccurate, deceptive or false information. Readers have an interest in knowing the identity of writers in order to better assess their credibility or authority.^17^ Knowing that an author of an economic blog does not have any formal training in economics, for example, might make readers question her advice more than they would otherwise. Anonymity can obstruct verifiability.

In some cases, anonymity can help the dissemination of extremisms, including terrorism. Anders Breivik, who murdered 77 people in Norway in July 2011, wrote his 1,516‐page online manifesto under a pseudonym, Andrew Berwick. Anonymity can also facilitate other illegal activities, such as the publishing and consumption of child pornography.

More commonly, anonymity is often involved in online harassment. Both the physical distance involved in virtual interactions, in which people cannot look into each other's eyes, and the possibility of being anonymous, are thought to contribute to disinhibiting negative behaviour.^18^ A particularly disturbing element of being harassed online is that the victim cannot know the source of the threat – whether it is a neighbour who might have physical access to her, or a teenager thousands of kilometres away who is merely playing a prank – and therefore has little way of assessing its credibility.^19^

There are at least two reasons, however, why identifiability might not be successful in fighting online abusive speech. First, trolling – posting disruptive or inflammatory comments and harassing people online – has been found to be associated with sadism, psychopathy, Machiavellism and narcissism.^20^ Given that psychopathic individuals have deficiencies detecting and responding to threats,^21^ it is not clear that trolls will react positively to threats of being held accountable through identifiability. The scientific literature on trolling, however, is still not sufficiently developed for it to be certain that destructive behaviour online is mainly attributable to personality traits. Researchers have found that ordinary people can be influenced to engage in trolling by factors such as negative moods and the context of a discussion.^22^ Some software engineers like David Auerbach have emphasised design flaws in platforms such as Twitter that stress conflict – for example, a focus on individuals, rather than community, and the infinite threading of open conversations.^23^

The second reason why identifiability may not be the solution to online abuse is that, in the context of highly controversial online debates, identifiability may increase people's credibility and online popularity, which can encourage trolls to continue behaving nastily. A study that looked at more than 500,000 comments from around 1,600 online petitions on a German platform found that non‐anonymous individuals were more aggressive than anonymous ones.^24^

## 2 Choosing Between Anonymity and Identifiability

Anonymity and identifiability are typically not sought as aims in themselves. We only value them to the extent that they advance other – more important – aims, such as privacy, free speech, verifiability, and accountability. In this section, I weigh two options against each other: practicing identifiability while trying to achieve the benefits of anonymity through means different from anonymity, and practicing anonymity while trying to obtain the benefits of identifiability through other means. The objective is to consider ways of attaining our most important aims through some means other than anonymity and identifiability, with the motivation of obtaining the benefits they can bring without suffering their disadvantages.

Let us first consider the possibility of practicing identifiability and replacing anonymity. At its best, anonymity allows the expression of views without agents having to fear repercussions. Anonymity, however, is not the only way to achieve this end.

Regarding the expression of unorthodox views, it is possible – in theory – to have an open and free society in which people are interested in hearing opinions different to their own, and in which dissenters are not punished in any way for falling outside the mainstream. Even though this is a logical and metaphysical possibility, it is likely too utopic to be feasible. It would require cultural changes that cannot be brought about solely through institutional policies. It may even turn out that it is not psychologically possible for human beings as a species to embrace differences across the board. Of course, some societies are more open than others, and nearing that ideal through culture and socialisation can make a big difference to dissenters; but even in the most advanced societies, dissenters cannot be guaranteed to escape social repercussions.

Legal measures are a crucial complement in protecting speakers from bad consequences. But, again, even the best laws cannot fully protect speakers from repercussions. Laws can ban discrimination, for example, but proving that someone was denied a job because of discrimination is difficult. Employers can always appeal to excuses to justify their decision. In other cases, laws have limits because we want them to be limited; we do not want an oppressive state overregulating behaviour. Thus, laws can prohibit overt harassment, but they cannot ensure that people will not shun others for their views, as citizens do not have a legal duty to be friends with anyone. Social rejection, however, can be as damaging to someone's wellbeing as physical harassment.

In online settings, moderation can go a long way towards encouraging and maintaining constructive and high‐quality interactions.^25^ But moderation in the context of identifiability is also limited. It can protect users who have expressed a view from being attacked within the website in which the debate is taking place. But it can do nothing to protect people who can be identified and targeted outside of the moderated platform. Thus, an online forum can protect the author of an article from insults in the comments section by banning profane language, but it cannot stop trolls from harassing the author in Twitter or other contexts.

If we choose to practice identifiability, the available alternatives to bring about the benefits of anonymity, then, are not very promising. Socialisation, laws, and moderation have a limited power to avoid undesirable repercussions for speech.

Let us now consider the alternatives to identifiability if we choose to practice anonymity instead. At its best, identifiability allows for verifiability and accountability: by being correctly identified as the source of speech, speakers' qualifications and expertise can be assessed, people who act in praiseworthy ways can be commended, and wrongdoers can be reprimanded as appropriate. Presumably, however, the opportunity for admonition is more important than the chance to praise someone. Given that praise is often desired and sought after, the anonymous person can always disclose her identity (as long as there is proof of authorship) and reap the benefits of positive feedback. The main problem with lack of identifiability, then, is that we are not able to reproach wrongdoers.

Here again, moderation has an important, if limited, role to play. By banning and deleting unacceptable content, online platforms can obviate or minimise the need to punish or criticise people who misbehave online. The advantage of moderation is that it prevents harm. When it works well, no one gets threatened or harassed. The downsides include its cost (at present we do not have good enough algorithms to detect all harassment, so we need people to assess content), the lack of public accountability for wrongdoers (speakers of unacceptable views will not be made to face their peers), and the danger of unduly limiting speech.

With moderation, there is the further question of who may decide what is an unacceptable speech. It can be argued that platforms such as Twitter should be able to decide what kind of speech (if any) is banned on their websites, given that they are private companies and that individuals are not forced to use their services. As an analogy, we have no qualms about restaurants deciding on their own menus (as long as the options are safe). But social media is different. It is users who supply most of the content on these platforms. Diners at restaurants do not bring their own food. Furthermore, while there is typically a range of restaurants available for diners to choose from, platforms like Facebook and Twitter have become irreplaceable. Largely, what makes them so successful is that most people we know are on them. Their success has metaphorically transformed these platforms from restaurants where small groups of people gather, into public arenas where netizens meet to participate in society. Given that they have become somewhat indispensable to fully participate in one's society, Facebook and Twitter have a responsibility to be as inclusive as possible, because being excluded from these public forums can be significantly harmful. The importance of these platforms for individuals speaks against placing the power of allowing and banning speech (and speakers) in the hands of private companies that do not necessarily have the public interest as their priority.

Moderation, then, seems to be both too strong and too weak a measure. It is too strong because it is likely to engage in some undue censorship. Online platforms might favour being on the safe side, thus incurring false positives by censuring posts that should not be prohibited. In 2016, for instance, Facebook censored an iconic photograph from the Vietnam War because it included child nudity. The company subsequently revised its decision and agreed to allow the photograph after Norway's largest newspaper published an open letter of complaint to Mark Zuckerberg. If speech is unduly curtailed, society will be worse off. But moderation is also too weak a measure. First, because, so far, it has not been enough to hinder trolls on social media like Twitter and Facebook. Second, because, as has been mentioned, it can only be limited to specific platforms. Anyone can open their own blog or website. There will always be a venue for unacceptable discourse such as hate speech – even if it is not expressed on the most popular platforms.

If lack of identifiability is to be total – in other words, if we choose full anonymity as our standard – then moderation seems like the only possible option to reduce harms caused through speech online. Even then, moderation has its own problems, and cannot provide all the advantages of identifiability because it cannot offer accountability – at most, wrongdoers will not be able to express their views on mainstream online platforms, but they will never be held responsible for them – at least not publicly. Furthermore, full anonymity – in which absolutely nothing is known about the writer and there is no way to track her past activities – precludes verifiability (e.g. assessing the author's credentials), without there being any kind of tool, such as moderation, to substitute for identifiability.

### 2.1 The Problem

In a nutshell, as Alfred Moore puts it, the tension in the conflict between anonymity and identifiability lies in the contradictory normative positions that ‘anonymity is valuable because it enables expression free from fear of repercussions’ and that ‘anonymity is destructive because it enables expression free from fear of repercussions.’^26^ More specifically, we want to make sure both that valuable speech can be made to be low‐cost (have no repercussions), and that abusive and inappropriate speech is either not allowed to take place or if it does take place, that it is costly for the perpetrators; all that without resorting to regulation so heavy as to curtail free speech. If we choose either full anonymity or identifiability, it seems that we cannot achieve these goals.

## 3 Instituting Costs

Even the most liberal societies limit speech. What is controversial is not that there be limits to what we can say, but where the boundaries are drawn, what is the penalty for transgressing them and who may decide these matters (this third issue is largely beyond the scope of this article).

When it comes to anonymity and the value of speech, on one side of the spectrum, there is speech from which society benefits and that we want to incentivise, but that people may be discouraged from engaging in if their identity is not protected because of risks of harm involved. An example is participating in surveys about public health. In these kinds of contexts, it is desirable, whenever possible, to set up low‐cost situations (free from repercussions) to encourage participation through guaranteeing anonymity.

In the middle of the spectrum there is speech from which society largely benefits, but about which there are grey and contentious areas. Examples include political debate and protest. Liberal democracies benefit from having people exchange political ideas, arguments and sentiments, but there is much room for abuse (from manipulation, bad faith, and fake news, to intimidation and harassment), and there are many areas of controversy (e.g. where to draw the line between a heated defence of a view and an offensive one). In these kinds of contexts, one way to regulate discourse is to introduce costs for speech.

One option for introducing costs is having some degree of identifiability that forces people to face criticism from their peers. If people want to voice some view in a public forum, they should be willing to receive feedback. Another way of introducing costs is establishing penalties for trespassing limits. Penalties can vary in kind: they can be financial, reputational, or they can include limits to access, among others. If a netizen engages in trolling on Twitter, for example, his account may be suspended (temporarily or permanently, depending on the gravity of the wrongdoing).

On the other side of the spectrum there is speech that is very destructive and about which there is a broad societal agreement. In these cases, it is best to put policies in place that prevent such speech altogether, and establish high penalties for when prevention fails. There is nothing to be gained by death threats, for example: they do not make an argument, they are not an attempt to advance truth, they do not inform the public, nor do they promote positive social cooperation, and the psychological damage to the victim can be significant. Death threats are so serious that, above and beyond other kinds of online penalties, offenders should face legal consequences.

So far, it has been established that the problematic tension between anonymity and identifiability lies in the desirability of having no costs for some kinds of speech and costs for other kinds; if we choose full anonymity or identifiability, we have either low or high costs in *all* situations. In other words, we do not want it to be equally costly to say anything at all – some assertions in some contexts should be more costly than others, but neither full anonymity nor identifiability allow for that nuance. Happily, anonymity and identifiability are not binary options. There is a third possibility that, although technically a form of anonymity, lies as a middle way: pseudonymity.

## 4 Pseudonymity

Derived from the Greek ‘false name’, pseudonymity involves the identification of an author through a tag that does not correspond to her real name. Pseudonymity does not amount to full identifiability because, while a pseudonym allows the identification of an author as one and the same author across time and publications in one or more platforms, it does not allow others to link the author's pseudonym to her real name or identity. Although pseudonymity is a subtype of anonymity, as it can block the link between authorship and identity, the veil of invisibility is thinner than in full anonymity, because the author who publishes under a single pseudonym has an identifiable persona that can be stable and held accountable for her actions. When pseudonyms are used more than once, pseudonymity is a means for achieving a degree of anonymity that is short of full anonymity.

In his compelling defence of a fictionalist account of pseudonymity, Lloyd Humberstone argues that when ‘X is a pseudonym, X is a fictional character’.^27^ What makes pseudonyms different from nicknames is that they function as an alternative identity – a fictional character, an alter ego, a persona – in a way that nicknames do not. That is why we can say that ‘Lewis Carroll was Charles Dodgson’, but we would not say that ‘Ike was Dwight Eisenhower’. In the latter case, it would be more natural to say that ‘Eisenhower was *called* Ike’. In contrast, Dodgson was not called Lewis Carroll by those around him, and whenever strangers made that mistake, he was upset by it and corrected them in their error, as he did not always want to acknowledge his alter ego.^28^ Pseudonymity differs from the anonymity that strangers enjoy in a crowded street in that it can hide true identity through acting like a mask that becomes the identifier of an alter ego.

Pfitzmann and Hansen have classified pseudonyms into three categories, in order of strength of anonymity.^29^ *Public pseudonyms* (the weakest in anonymity) are those for which the link between a persona and the person's identity may be publicly known (e.g. if there is a public directory linking pseudonyms with real names). *Non‐public pseudonyms* are those that can be linked to a person's identity by certain privileged parties, but not by the public at large. *Unlinked pseudonyms* (the strongest in anonymity) are those that are only known by the holder of the persona; those for which the link is not meant to be found out by anyone else. In this article, I am only interested in non‐public and unlinked pseudonyms, as public pseudonyms are almost equivalent to full identifiability, acting more like nicknames than proper pseudonyms.

Unlinked pseudonyms render speech low‐cost. They are thus advisable in contexts:

- so sensitive that people will be reluctant to express themselves if their identity is not heavily protected, provided these kinds of speech are desirable (i.e. society is worse off without them);
- in which moderation is feasible, and therefore the leeway for abuse is low

Examples of such contexts are sexual health forums. The context is so sensitive that people might not seek help or ask a question if their identities are not protected. But society is better off if people can ask questions about their health. In such forums, moderation is feasible: moderators can review the questions being asked before posting them. Controversy as to what ought to be banned is likely to be manageable: anything that is not a medical question is not meant to be in the forum. Verified physicians could provide answers using their real names.

Non‐public pseudonyms are more appropriate for more open forums of discussion, such as newspapers, political blogs, and platforms like Twitter. Within non‐public pseudonyms, there is a range of options to regulate levels of anonymity and costs. The more restricted and stable the pseudonym, the more costly the speech can be.

Suppose an international organisation, Pseudo, were in charge of distributing pseudonyms for online activities. They would act as fiduciaries of the link between real names and pseudonyms. Suppose further that netizens were allowed only two or three pseudonyms in their lifetime, to be used as they please (people could use them simultaneously in different platforms, or only use one across all platforms for years, and subsequently use the others). Only pseudonyms approved by Pseudo would be able to participate in online platforms like Facebook, Twitter, and YouTube, and to open websites of their own. Authenticity could be verified through technologies such as zero‐knowledge proof or blockchain. If there were a serious crime committed, such as a death threat, Pseudo could reveal the identity of the criminal to the police. If a pseudonym was abused by using hate speech, for example, the bearer might lose the privilege of using that pseudonym in the future (perhaps after one warning, or immediately if the infraction were grave enough). If someone were to lose all of her pseudonyms, she would be forced to either not participate on online debates or participate using her own name.

It could be argued that the penalty for transgression should be identifiability, as it is the only way to achieve accountability. But identifiability for any kind of transgression would undermine the whole system of pseudonymity. It would be too high a penalty because it would make people afraid of speaking out. Identifiability means they could lose their friends, their job, and even risk their life for saying the wrong thing. Having repercussions (in the form of feedback from peers), penalties other than identifiability, as well as the possibility of revealing people's identity to the police if they commit a crime, amounts to having a significant degree of accountability. Furthermore, there will be grey areas in which it will be controversial whether someone committed an infraction. Categorising speech is not always easy, and it is better to have a kind of penalty that gives people a second chance.

It could then be objected that perhaps the ‘two strikes and you're out’ penalty is too high for a punishment. In face‐to‐face communications, one is rarely locked out from the possibility of further communication. In some occasions, a person can say something so insulting that the offended party may never want to speak to her again. But, even then, the offender does not lose the opportunity to participate in speech in her wider community. It should be noted, however, that under the system I am proposing, offenders could still participate in online speech, but they would be forced to use their own names if they have lost their pseudonyms. If we were to find that too many people were losing their pseudonyms for contestable transgressions, then we could adjust the severity of the penalty by imposing a time‐out, rather than a permanent loss of pseudonyms (similar to driving licence suspensions after committing a certain number of infractions).

My main argument in this article is that pseudonymity can be used as a tool to regulate the costs of speech to make the online world a better place; exactly how we carve up this tool remains up for discussion, will vary depending on context, and should develop as we gain more experience. How much stability and restriction are needed to keep the Internet relatively free from harassment is an empirical question in need of further research. It may be that to tackle online harassment, it will be necessary to have a unique lifetime pseudonym across all mainstream platforms like Facebook and Twitter. Perhaps that option is compatible with having multiple pseudonyms in other types of websites in which abuse is less likely to occur.

Content moderation and the management of pseudonyms are likely to be easier if there are different platforms designed for different kinds of discourse; a platform for political discussions, another for discussions on arts and media, another for personal interactions, and so on. If we restrict the content, then it will be easier to strike the right balance between anonymity and identifiability, to choose the right kind of pseudonymity for every platform on the basis of the risk of abuse and the value of the free speech being exercised, and to moderate.

Philosophers Francesca Minerva and Michael Tooley have argued (separately) that a sensible response in academia to the threat of online exposure to trolls, which is resulting in self‐censorship, is to allow researchers the possibility of publishing under pseudonyms on academic journals. A central and secure website would be used to register one's unique pseudonym. Academics wanting their pseudonymous publications to be considered in a job application or similar work decisions could provide the relevant committee with a one‐time password to the central website that would allow the committee to verify the author's work.^30^ Professional watchdogs could also have access to identities and pseudonyms, under a duty of confidentiality, to make sure there were no abuses such as undisclosed conflicts of interest.^31^

With non‐public pseudonyms in general, someone in a position of responsibility has to have the possibility of accessing the relation between pseudonyms and identities. This would be a fiduciary relationship in which netizens must be able to trust that their identities will not be unveiled unless they transgress the established rules. It would be better if the institutions involved in the management of pseudonyms were supranational so that political dissenters may be better protected from repressive governments. Information about real identities would only be passed on to the relevant authorities when there is broad international consensus that the offence being committed should be considered a crime, or is a violation of human rights, and a severe one at that. An example would be child pornography.

It could be objected that reliance on trusted experts and supranational mechanisms of enforcement makes this proposal unfeasible. The existence of the Internet Corporation for Assigned Names and Numbers (ICANN), a non‐profit organisation that coordinates the maintenance and procedures of databases related to the namespaces of the Internet, and in which more than 100 governments are represented, shows that it is possible, even if not easy or perfect.

## 5 The Question of Deception

If pseudonyms are names that refer to fictional characters, questions of deception arise. First, there is the deception involved in pretending to be someone else. Second, it is likely that netizens will engage in more deception in the content of what they say if there are no repercussions to their true identities.

The first kind of deception can be dealt with easily by tagging pseudonyms as such. Netizens could always know whether they are engaging with a real identity or an alter ego. In some contexts, such as academic journals, it will be important to have a system of verification whereby academics can have their credentials certified. A reader would not know the real name of the author, but she could be confident that the author has a PhD in Philosophy, say, which would reinstate some of the authority granted to speech by identifiability. Similar systems of verification would also help in minimising deception of the second kind. If academic publications might be verified eventually (by a hiring committee, for example or professional watchdogs, as Minerva suggests), authors will have an incentive to be truthful and rigorous. In opinion forums, we could allow some flexibility: people who would like a part of their identities verified by Pseudo could have that. For example, if a woman wanted to have her gender verified and have it as part of her online alter ego, that might give her more authority when giving an opinion about what it is like to be a woman.

Unlinked pseudonyms will be riskier when it comes to deception. With unlinked pseudonyms, people will not be able to appeal to any kind of authority to support the standing of their speech. It may be that readers in such contexts will be more aware of the possibility of deception by authors, and therefore more wary of what they read. It may also be that, when authority cannot be claimed, the force of arguments and references to empirical evidence will gain in importance.

If pseudonyms were broadly implemented across platforms, the Internet would become a masquerade ball. Wearing a mask is not a deceptive act. It hides one's identity, but it does so forthrightly.

## 6 The Question of Censorship

A tempting objection to my proposal of regulating speech through the introduction of costs is that such methods amount to censorship. I disagree. Just as the prohibition of killing does not amount to repression, prohibitions against aggressive behaviour like hate speech and revenge porn do not amount to censorship.

The question becomes thornier when it comes to the expression of unpopular views that are legal but considered politically incorrect by some and utterly unacceptable by others. Consider the example of arguing that animals are as morally worthy as young infants. Many people (perhaps even most) might find this view repugnant. But if the thought is expressed in the form of an argument, with premises and conclusions, in language free from insults and profanities, and without inciting to violence, it is not illegal speech.

This kind of unpopular speech would be possible, according to my proposal, but it would have a price to pay – at a minimum, the speaker of such a view would be the bearer of a stable online persona through which he would be forced to face criticisms by his peers. It is quite likely that the criticism faced would be substantial and unpleasant to receive. His online life in the platform used may be negatively affected by the expression of his views – perhaps other netizens will not engage with him in positive interactions that would have otherwise taken place had he not expressed this view. However, the impact of the backlash he may receive will be significantly contained and diminished by his pseudonym. If he has access to other pseudonyms, he can occupy those other personae whenever he gets tired of answering objections to his argument. More importantly, his offline life is safe: his work and family environment can remain untouched by the online controversy.

My proposal makes the expression of unpopular views a costly affair, but not so costly that people do not have a reasonable option to speak up. Voicing highly contentious views can amount to a kind of civil disobedience. For most of history, the view that women deserve equal rights to men was considered outlandish. Back then, expressing this opinion could have brought trouble to the speaker. Given that people are generally aware of what the norms in their society are, voicing unpopular views about which one holds strong convictions can end up amounting to a kind of sacrifice for the good of society, for a better future. Eventually, enough people might listen to the controversial view, be convinced by it, and voice it themselves, until it becomes part of the norm.

Social norms are useful: they provide structure to human interactions and help us socialise people into the culture we want to live in. For social norms to work as such, people have to feel some amount of resistance when they break the norms, as when they defend views that are broadly considered unacceptable. But social norms must not be too rigid. To allow for positive social change and the evolution of views, deviating from social norms should not be too costly. Pseudonymity can be a tool that allows us to fine‐tune the cost of divergent speech online. In instances in which we, as a society, are fairly certain that our norms are correct, such as the case of death threats, speech should be very costly (e.g. causing one to lose the privilege of anonymity, facing legal consequences); in cases that inhabit grey zones, such as the debate about the moral status of animals, speakers should be made to face criticism, but in a way that does not make it too costly to express a defendable view that may be widely accepted in the future, thanks to speakers like them.

## 7 The Importance of Contracts for Protecting Identity

In the search for striking the right balance between anonymity and identifiability, mistakes can be made. Societies can change their minds about what the right amount of anonymity should be in different contexts. But anonymity is a kind of contract: guardians of anonymity are supposed to protect the identity of people as long as the latter stick to their side of the deal. Pseudonymity can only work long‐term if that promise is kept. If online platforms give people reason to distrust them, pseudonymity will not be able to perform its function of incentivising free speech.

Today, Internet companies often include a proviso in their terms and conditions that ‘they can unilaterally change their terms of service agreement without any notice of changes to the users’.^32^ As long as companies can get away with changing their terms as they please, pseudonymity will be too frail to support free speech.

It may be that we need to experiment with different pseudonymity contracts before we find what works best for different online platforms. Those experiments should be clearly labelled as such. Once a decision is made and a contract arrived at, it should be legally binding. If adjustments are made to the pseudonymity contract, they should only apply to new users and old users who prefer the new agreement. If some changes are unavoidable and must apply to all users (e.g. to comply with new legal regulations), old pseudonyms must not be unveiled and new pseudonyms must be issued for old users who want them.

A contract is a serious commitment; if broken, it erodes trust and breaks down institutions, practices, and social cooperation. Pseudonymity can enable people to say what they would not otherwise say, but only if institutions can be trusted to have the necessary commitment and competence to protect identities.

## 8 Conclusion

In this article I have argued for the use of pseudonyms as a promising tool to face the challenges to free speech in the online world. Pseudonymity may not be appropriate for all speech contexts (e.g. speech by politicians on a political campaign), but it may be suitable for many, if not most, contexts online in which people interact and express their opinions. Pseudonymity is certainly not the only or most important tool for creating constructive dialogues online. In order to enjoy harmonious societies, it is vital that we improve social conditions through education, equality, justice and culture. These tools may do more to reduce harassment and repression than any pseudonymity tool. Nonetheless, even the most democratic, equal, and just of societies would benefit from implementing pseudonymity online, as people will feel freer to explore bold ideas if their identities are protected from harsh consequences.

Democracies cannot afford to continue with the current trend online regarding free speech. We are allowing repressive governments to surveil and silence activists and political dissenters. We are failing to protect vulnerable populations against abuse. Journalists are being intimidated. Academics are self‐censoring out of fear. Online trolling is putting women off politics.^33^ Valuable speech is losing out. Voices that would contribute to our societies are being lost, drowned out by vitriolic speech. Some of the Internet's faults stem from it not having been designed to be the mass medium it has become. We have to redesign it to better serve us. Implementing pseudonymity along the lines of my proposal would be a good start.

## Notes

1 Justin McCurry, ‘Japanese blogger stabbed to death after Internet abuse seminar’, *The Guardian* (26 June 2018).

2 Gareth Corfield, ‘Arrests for “offensive” Twitter and Facebook messages up by a third’, *The Register* (2 June 2016).

3 William Makepeace Thackeray, *Roundabout Papers* (Project Gutenberg, 2018).

4 Kathleen A. Wallace, ‘Anonymity’, *Ethics and Information Technology* 1,1 (1999): 21–31, at p. 29. Wallace has categorised anonymity into three general purposes:

1. ‘anonymity for the sake of furthering action by the anonymous person, or agent anonymity;
2. anonymity for the sake of preventing or protecting the anonymous person from actions by others, or recipient anonymity;
3. anonymity for the sake of a process, or process anonymity.’

Though Wallace's categories are coherent, I have opted for the more economical classification of anonymity for the sake of protecting agents and furthering actions (which includes both desirable processes and actions by protected agents). Consider blind peer review. The reasons for anonymity can be fully captured by the objectives of protecting agents (authors and reviewers) and furthering desirable actions (the publication of research, both for the sake of the author and of society).

5 Jack Grove, ‘Turkey's persecution of academics is ‘‘unmatched’’’, *Times Higher Education* (14 June 2017).

6 Eric Barendt, *Anonymous Speech: Literature, Law and Politics* (Oxford: Hart Publishing, Kindle Edition, 2016), Loc 313.

7 Barendt op. cit., Loc 1335.

8 Barendt op. cit., Loc 407.

9 Virginia Woolf, *A Room of One's Own* (Harmondsworth: Penguin, 2000), pp. 50–51.

10 Angelo Corlett, J., ‘Ethical issues in journal peer‐review’, *Journal of Academic Ethics* 2,4 (2005): 355–366.

11 Quoted by Barendt op. cit., Loc 2191.

12 John Stuart Mill, *On Liberty, Utilitarianism, and Other Essays* (Oxford: Oxford University Press, 2015), p. 19.

13 Thomas Scanlon, ‘A theory of freedom of expression’, *Philosophy and Public Affairs* 1,2 (1972): 145–165.

14 James M Jasper, *The Art of Moral Protest* (Chicago, IL: The University of Chicago Press, 1997), pp. 130–50.

15 Jasper op. cit., pp. 367–80.

16 Plato, *Republic* (Indianapolis, IN: Hackett Publishing, 1992), 2:359a–2:60d.

17 Barendt op. cit., Loc 415.

18 Noam Lapidot‐Lefler & Azy Barak, ‘Effects of anonymity, invisibility, and lack of eye‐contact on toxic online disinhibition’, *Computers in Human Behavior* 28,2 (2012): 434–443.

19 Barendt op. cit., Loc 3979.

20 Erin E. Buckels, Paul D. Trapnell & Delroy L. Paulhus, ‘Trolls just want to have fun’, *Personality and Individual Differences* 67 (2014): 97–102.

21 Sylco S. Hoppenbrouwers, Berend H. Bulten & Inti A. Brazil, ‘Parsing fear: A reassessment of the evidence for fear deficits in psychopathy’, *Psychological Bulletin of the American Psychological Association* 142,6 (2016):573–600.

22 Justin Cheng *et al*. ‘Anyone can become a troll: Causes of trolling behavior in online discussions’, Paper presented at the Proceedings of the 2017 ACM Conference on Computer Supported Cooperative Work and Social Computing, 2017.

23 David Auerbach, ‘Twitter is broken’, *Slate* (7 October 2016).

24 Katja Rost, Lea Stahel & Bruno S. Frey, ‘Digital social norm enforcement: Online firestorms in social media’, *PLoS One* 11, 6 (2016): e0155923.

25 James Grimmelmann, ‘The virtues of moderation’, *Yale Journal of Law and Technology* 17,1 (2015): Article 2.

26 Alfred Moore, ‘Anonymity, pseudonymity, and deliberation: Why not everything should be connected’, *The Journal of Political Philosophy* 26,2 (2018) 169–192, at p. 169.

27 Lloyd Humberstone, ‘Names and pseudonyms’, *Philosophy* 70 (1995): 487–512, at p. 489.

28 Humberstone op. cit., p. 497.

29 Andreas Pfitzmann & Marit Hansen, ‘A terminology for talking about privacy by data minimization: Anonymity, unlinkability, undetectability, unobservability, pseudonymity, and identity management’ (version v0.34, 2010), available at: https://dud.inf.tu-dresden.de/literatur/Anon_Terminology_v0.34.pdf.

30 Michael Tooley, ‘Solutions to the new threats to academic freedom?’, *Bioethics* 28,4 (2014): 163–165.

31 Francesca Minerva, ‘Why publishing pseudonymously can protect academic freedom’, *Bioethics* 28,4 (2014): 178–180.

32 Logan Koepke, ‘“We can change these terms at anytime”: The detritus of terms of service agreements’, *Medium* (18 January 2015).

33 Gemma Ryall, ‘Online trolling putting women off politics, says union’, *BBC News* (20 May 2017).

